# Trends in bullying victimization in Scottish adolescents 1994–2014: changing associations with mental well-being

**DOI:** 10.1007/s00038-017-0965-6

**Published:** 2017-03-15

**Authors:** Alina Cosma, Ross Whitehead, Fergus Neville, Dorothy Currie, Jo Inchley

**Affiliations:** 1 School of Medicine, Child and Adolescent Health Research Unit (CAHRU), Medical and Biological Sciences Building, University of St Andrews, St Andrews, Scotland, UK; 2 School of Psychology and Neuroscience, University of St Andrews, St Andrews, FifeKY16 9TF Scotland, UK

**Keywords:** Bullying victimization, Mental well-being, Happiness, Confidence, Time trends

## Abstract

**Objectives:**

Bullying victimization among schoolchildren is a major public health concern. This paper aims to analyse the changing associations over two decades between bullying victimization and mental well-being in a representative Scottish schoolchildren sample.

**Methods:**

Data were collected in six rounds of the cross-sectional Health Behaviour in School-aged Children study in Scotland, with 42,312 adolescents (aged 11, 13 and 15 years). Logistic and linear regressions were used to examine changes in the association between bullying victimization and mental well-being.

**Results:**

The prevalence of bullying victimization rates in Scotland increased between 1994 and 2014 for most age–gender groups, apart from 13-year-old boys and 15-year-old girls. Over time, female victims reported less confidence and happiness and more psychological complaints than their non-bullied counterparts. This worsening effect over time was not observed in boys.

**Conclusions:**

Overall, our evidence indicates that the associations between bullying victimization and poor mental well-being strengthened overtime for bullied girls. This finding might partly explain the observed deterioration in mental health indicators among Scottish adolescent girls.

**Electronic supplementary material:**

The online version of this article (doi:10.1007/s00038-017-0965-6) contains supplementary material, which is available to authorized users.

## Introduction

Bullying victimization is a common problem in schools worldwide (Smith et al. 2016; Elgar et al. 2013). These incidents can be defined as situations where victims are exposed to repeated, targeted and deliberately unkind actions, where they are unable to defend themselves (Solberg and Olweus 2003). The prevalence of bullying victimization shows wide variation depending on age, gender, measurement used or country (Smith et al. 2016; Molcho et al. 2009). For bullying perpetration, there is a clear cross-cultural gender and age pattern with boys more likely to be perpetrators than girls and decreasing prevalence with age. For bullying victimization, however, the situation is less consistent. According to the latest Health Behaviour in School-aged Children (HBSC) International Report, gender differences (boys being more likely than girls to indicate they have been bullied at least 2–3 times in the last couple of months) were seen only in a minority of countries in Europe and North America (Inchley and Currie 2016). This report indicates wide cross-national variation in the prevalence of being bullied with rates ranging from 2 to 35%. Despite such variation in prevalence, there is a universal decline in bullying victimization with increasing age (Inchley and Currie 2016; Smith et al. 1999). Nonetheless, for those who do experience bullying, there can be long-term impacts. For example, a recent meta-analysis highlights that victimisation in adolescence is a stronger determinant of future victimisation than is experience of bullying in earlier childhood (Pouwels et al. 2016).

There is currently inconsistent evidence regarding changes over time in bullying victimization among young people. Looking at trends in bullying victimization across 30 European countries, Chester et al. found a decrease from 2002 to 2010 in occasional and frequent victimization across a third of participating countries (Chester et al. 2015). However, in the majority of countries included in this study, the prevalence did not change significantly during this time. Moreover, bullying victimization rates are reported to have declined in Finland (Ilola and Sourander 2013; Santalahti et al. 2008), Germany (Melzer et al. 2012), US (Perlus et al. 2014), Italy (Vieno et al. 2015) and Romania (Cosma et al. 2015) but increases have been reported in other countries such as Greenland (Schnohr and Niclasen 2006) and US (only older adolescent girls) (Kessel Schneider et al. 2015).

Cross-sectional and longitudinal evidence indicates a strong association between bullying victimization and mental health problems. Victimization at school has been repeatedly linked to greater levels of internalizing problems (Reijntjes et al. 2010), feelings of loneliness (Juvonen et al. 2000), negative affect (Dill et al. 2004) and lower levels of self-esteem and negative self-perception (Boulton et al. 2010). A large number of longitudinal studies also point out that these negative effects extend to later adolescence and young adulthood (Takizawa et al. 2014; Zwierzynska et al. 2013), especially in the case of depression (Ttofi et al. 2011).

Some studies suggest that age and gender might act as moderators in the interplay between bullying victimization and mental well-being. Although some studies conclude that both boys and girls are equally likely to experience internalizing problems (Kendrick et al. 2012), other research indicates that adolescent girls who are bullied might experience worse well-being outcomes than boys (Siegel et al. 2009). Trying to explain this gender effect, Hankin and Abramson (2002) suggest that girls might be more likely to suffer from the psychological repercussions of bullying because they tend to attribute peer victimization to perceived social deficiencies.

In Scotland, bullying prevention among school-aged children is a priority of national and local governments. A new national approach was proposed in 2010 by the Scottish Government which aims to promote a common approach to bullying prevention and to ensure that work across all agencies and communities consistently and coherently contributes to reducing bullying (Scottish Government 2010). Moreover, charities, together with national educational reforms, have worked together to reduce school violence and to promote a safe learning environment for all schoolchildren across the country (Scottish Government 2010). On the other hand, from 2012 the Scottish Government through the Getting It Right For Every Child (GIRFEC) national initiative has aimed to support a holistic approach to well-being in young people by offering ‘the right help at the right time’ from the right people (Scottish Government 2012, p5). Despite these initiatives, there has been a worsening of mental well-being among Scottish young people, especially 15-year-old girls (Black and Martin 2015; Cosma et al. 2016). A similar decline in mental health among adolescent girls, both in the UK and Europe, has been reported in previous studies (e.g. Collishaw 2015).

Whilst there are some studies looking at changes in bullying victimization over time, there is a lack of research into potential changes in the association between bullying victimisation and mental well-being over time, especially in countries such as Scotland where tackling bullying and promoting child and adolescent mental well-being have been part of the school curriculum ever since 2010. Using a nationally representative sample of Scottish schoolchildren, the present study aims to fill this evidence gap by exploring the impact of bullying victimization on adolescents’ mental well-being over two decades (1994–2014). Considering the wide age and gender variations identified in the research literature, the first aim is to analyse time trends in bullying victimization for 11-, 13- and 15-year-old Scottish boys and girls. Second, we investigate the change over time in the associations between bullying victimization and mental well-being for each age/gender permutation.

## Methods

Data from six rounds of the Scottish HBSC survey were used (1994–2014). HBSC is an international cross-sectional survey conducted every 4 years. Each survey year, a nationally representative sample of school-aged children aged 11, 13 and 15 complete a paper-based survey in a classroom setting (Currie et al. 2014). The study was granted ethical approval by the Moray House School of Education Ethics Committee, University of Edinburgh (survey rounds 1994–2010), and by the University of St Andrews Teaching and Research Ethics Committee (for the 2014 survey). Prior informed consent was obtained at local authority, school, parent, and pupil levels.

### Measures bullying victimization

An adapted version of the Dan Olweus (Olweus 1994) bullying questionnaire was used in every survey year. After reading a definition of bullying which emphasized the main characteristics (intentionality, power imbalance and repetition), participants were asked to indicate if they have been bullied in the past couple of months with the following response options: “I haven’t been bullied”, “1–2 times”, “More than 2–3 times a month”, “About once a week”, “Several times a week”. A dichotomized variable was created, which grouped pupils indicating a frequency of victimization of at least 2–3 times a month versus those that indicated a lesser frequency.

### Confidence

Every survey year participants were asked how often they feel confident in themselves with five response options (“Never”, “Hardly ever”, “Sometimes”, “Often”, “Always”). A binary variable was created, in which all the answers indicating a frequency of often and always were recoded to ‘1’ (high confidence), whereas all the other option responses were recoded to ‘0’ (low confidence).

### Happiness

Every survey year participants were asked how they feel about their lives at the moment, with four response options “I’m not happy at all”, “I don’t feel very happy”, “I feel quite happy”, “I feel very happy”. A binary variable was created, in which all the answers indicating a frequency of ‘quite happy’ and ‘very happy’ were recoded to ‘1’ (happy), whereas all the other option responses were recoded to ‘0’ (unhappy).

### Psychological complaints

Every survey year participants indicated the frequency with which they experienced each of eight health symptoms (nervousness, bad temper, feeling low, sleep difficulties, headaches, stomach aches, backaches and dizziness) in the past 6 months. The response options were “About every day”, “More than once a week”, “About every week”, “About every month”, “Rarely or never”. These items are a well-validated measure of subjective well-being (Ravens-Sieberer et al. 2008), which can either be used together by creating a score of multiple health complaints, or can form a bi-dimensional measure: somatic and psychological complaints. For the purpose of this study, we have used only the psychological complaints dimension (good internal consistency; *α* = 0.73) by summing up four items (nervousness, bad temper, feeling low, sleep difficulties), with higher scores (max. 16) reflecting higher levels of psychological complaints (Haugland and Wold 2001).

### Data analyses

The SPSS (v. 22) complex samples toolkit was used to adjust for clustering of pupils within local authority and school, each within survey year. Logistic regressions were conducted to examine the impact of survey year (treated as continuous) and school grade (treated as categorical) on bullying victimization (being bullied at least 2–3 times a month in the past couple of months). Analyses were stratified by gender as the nature of bullying victimization differs between boys and girls. Logistic and linear regression analyses were conducted to examine changes over time in associations between bullying victimization and categorical (confidence and happiness) and continuous (psychological complaints) mental well-being outcomes, respectively.

## Results

The overall sample included in the analysis consists of 37,658 pupils who reported on their experience of being bullied since 1994. The numbers for the outcome measures (confidence, happiness, and psychological complaints) are slightly lower due to missing data responses (see Table 1).

**Table 1:** Descriptive statistics by gender among Scottish adolescents

	Boys *N* (%)	Girls *N* (%)	Total *N* (%)
Age			
11-year-olds	6322 (34.1)	6329 (33.1)	12,651 (33.6)
13-year-olds	6162 (33.2)	6314 (33.1)	12,476 (33.1)
15-year-olds	6075 (32.7)	6454 (33.8)	12,530 (33.4)
Total	18,560 (100)	19,098 (100)	37,658 (100)
Survey year			
1994	2393 (12.9)	2539 (13.3)	4932 (13.1)
1998	2739 (14.8)	2848 (14.9)	5589 (14.8)
2002	2213 (11.9)	2151 (11.3)	4363 (11.6)
2006	2997 (16.2)	3099 (16.2)	6097 (16.2)
2010	3175 (17.1)	3280 (17.2)	6456 (17.1)
2014	5040 (27.2)	5177 (27.1)	10,218 (27.1)
Total	18,560 (100)	19,098 (100)	37,658 (100)
Bullying victimization			
Never	13,424 (72.3)	13,462 (71.1)	26,886 (71.4)
Once or twice	3201 (17.3)	3562 (18.7)	6763 (18.0)
2 or 3 times a month	825 (4.4)	905 (4.7)	1730 (4.6)
Once a week	478 (2.6)	460 (1.8)	939 (2.5)
Several times a week	629 (3.4)	707 (3.7)	1337 (3.6)
Total	18,560 (49.3)	19,098 (50.7)	37,658 (100)
Confidence			
Never	630 (3.4)	1145 (6.0)	1776 (4.8)
Hardly never	1222 (6.7)	2734 (14.4)	3956 (10.6)
Sometimes	4359 (23.7)	6513 (34.3)	10,872 (29.1)
Often	7878 (42.9)	6222 (32.8)	14,101 (37.7)
Always	4287 (23.3)	2368 (12.5)	6656 (17.8)
Total	18,379 (100)	18,984 (100)	37,363 (100)
Happiness			
I’m not happy at all	263 (1.4)	444 (2.3)	707 (1.9)
I don’t feel very happy	1117 (6.1)	1985 (10.5)	3103 (8.3)
I feel quite happy	8422 (45.1)	9322 (49.1)	17,644 (47.2)
I feel very happy	8736 (47.4)	7222 (45.3)	15,958 (42.7)
Total	18,439 (100)	19,975 (100)	37,414 (100)
Psychological complaints^a^	18,098 (4.03)	18,807 (5.08)	36,905 (4.56)

^a^ *N* (mean score)

### Trends in bullying victimization

Overall, 10.6% of all participants indicated that they had been bullied at least 2–3 times a month in the past couple of months (10.4% of boys and 10.9% of girls). Linear trend analyses revealed an overall small but significant increase in bullying victimization [*F* (1,1453) = 32.69, *p* < .001] between 1994 (when 10.4% had been bullied at least twice) to 2014 (13.8%) (OR 1.02, 95% CI 1.01–1.03). Results indicate that, between 1994 and 2014, a significant linear increase was observed for 11-year-old girls (11.8–17.1%) [*F* (1,1453) = 12.16, *p* = .001] and boys (11.5–15.8%) [*F* (1,1453) = 5.23, *p* = .02), 13-year-old girls (11.3–18.8%) [*F* (1,1453) = 16.80, *p* < .001] and 15-year-old boys (7.7–10.5%) [*F* (1,1453) = 5.41, *p* = .02] (Table 2). No change was seen for 13-year-old boys [*F* (1,1453) = 1.04, *p* = .31] or 15-year-old girls [*F* (1,1453) = 1.86, *p* = .172]. The steepest increase was observed for the 13-year-old girls (OR 1.03, 95% CI 1.02–1.05) A main effect of age emerged, such that the 15-year-olds (both boys and girls) reported the lowest prevalence compared with the other two age categories.

**Table 2:** Trends in bullying victimization among Scottish adolescents between 1994 and 2014 (%± 95 CI)

	Boys							Girls						
	1994 (%CI)	1998 (%CI)	2002 (%CI)	2006 (%CI)	2010 (%CI)	2014 (%CI)	Linear trends 1994 to 2014 OR ± 95% CI a	1994 (%CI)	1998 (%CI)	2002 (%CI)	2006 (%CI)	2010 (%CI)	2014 (%CI)	Linear trends 1994 to 2014 OR ± 95% CI^a^
11-year-olds	11.5 (9.4–13.9)	11.9 (9.9–14.1)	9.7 (7.9–11.8)	10.8 (8.7–13.3)	10.7 (8.8–12.9)	15.2 (13.0-17.6)	1.02 (1.00, 1.02)*	11.8 (9.3–14.8)	11.7 (9.9–13.8)	10.3 (8.3–12.7)	10.2 (8.3–12.4)	13 (10.5–16.1)	17.1 (14.7–19.7)	1.02 (1.01, 1.03)***
13-year-olds	13.3 (11.1–15.8)	10.5 (8.6–12.8)	9.6 (7.5–12.2)	10.6 (8.7–12.8)	10.9 (9.1–13.1)	13.5 (11.4–16.3)	1.00 (0.99, 1.02)^ns^	11.3 (9.0–14.0)	11.2 (9.3–13.3)	9.7 (7.8–12.1)	11.8 (10.0-13.9)	13.7 (11.4–16.3)	18.8 (16.1–21.7)	1.03 (1.01, 1.04)**
15-year-olds	7.7 (5.6–10.4)	5.6 (4.2–7.5)	4.9 (3.5–6.8)	6.9 (5.5–8.6)	8.2 (6.8–10.0)	10.5 (7.5–11.1)	1.02 (1.00, 1.04)*	6.8 (5.3–8.9)	5.9 (4.3-8.0)	6.3 (4.5–8.8)	6.7 (5.1–8.6)	4.5 (3.5–5.8)	8.9 (7.1–11.0)	1.01 (0.99, 1.03)^ns^

*p ≤ .05, **p ≤ .01, ***p ≤ .001,   *ns* non-significant

^a^Logistic regression (OR ± 95% CI)

### Trends in associations between bullying victimization and mental well-being indicators

The following analyses were stratified by age group and gender, and indicate heterogeneity in the association between bullying victimization and mental well-being over time. In the initial phase of the data analysis, all models were controlled for socioeconomic status and bullying perpetration (results not presented here). Since the results had the same pattern with and without the control variables, it was decided that they would be dropped from the main analysis. The main analysis for each of the three mental well-being indicators are summarized in Table 3 (see Supplementary Materials for accompanying figures).

**Table 3:** Associations between bullying victimization and mental well-being outcomes among Scottish adolescents between 1994 and 2014

	Boys			Girls		
	11-year-olds	13-year-olds	15-year-olds	11-year-olds	13-year-olds	15-year-olds
Confidence (OR ± 95% CI)^a^						
Survey year^b^	1.01 (1.00, 1.02)*	0.99 (0.98, 1.00)	0.97 (0.96, 0.98)***	1.02 (1.01, 1.03)***	0.98 (0.97, 0.99)***	0.97 (0.96, 0.98)***
Not bullied (Ref.)^c^	1	1	1	1	1	1
Being bullied	0.53 (0.39, 0.72)***	0.51 (0.37, 0.72)***	0.38 (0.25, 0.59)***	0.65 (0.49, 0.87)**	0.74 (0.53, 1.03)	0.74 (0.47, 1.17)
Survey year × not bullied (Ref.)	1	1	1	1	1	1
Survey year × being bullied	0.98 (0.96, 1.01)	0.97 (0.94, 1.00)*	1.01 (0.98, 1.04)	0.97 (0.95, 1.00)*	0.96 (0.94, 0.99)**	0.96 (0.92, 0.99)*
Happiness (OR ± 95% CI)^a^						
Survey year^b^	1.04 (1.02, 1.06)***	1.03 (1.01, 1.05)***	0.99 (0.97, 1.00)	1.04 (1.02, 1.06)***	1.02 (1.00, 1.04)*	0.99 (0.98, 1.01)
Not bullied (Ref)^c^	1	1	1	1	1	1
Being bullied	0.27 (0.18, 0.40)***	0.24 (0.17, 0.35)***	0.26 (0.15, 0.44)***	0.29 (0.21, 0.42)***	0.34 (0.24, 0.48)***	0.44 (0.28, 0.70)***
Survey year × not bullied (Ref.)	1	1	1	1	1	1
Survey year × being bullied	0.97 (0.94, 1.00)	0.98 (0.95, 1.01)	1.00 (0.96, 1.03)	0.99 (0.96, 1.02)	0.97 (0.94, 0.99)*	0.96 (0.92, 0.99)*
Psychological complaints (B ± 95% CI)						
Survey year^b^	−0.07 (−0.01, −0.09)***	−0.02 (−0.04, −0.01)**	−0.04 (0.02, 0.06)***	−0.07 (−0.01, −0.09)***	0.00 (−0.02, 0.02)	0.09 (0.07, 0.11)***
Being bullied^c^	2.33 (1.74, 2.92)***	2.89 (2.18, 3.59)***	2.34 (1.40, 3.28)***	2.03 (1.42, 2.64)***	2.04 (1.36, 2.73)***	1.81 (1.06, 2.56)***
Survey year × being bullied	0.04 (−0.01, 0.09)	−0.01 (−0.07, 0.05)	0.01 (−0.06, 0.08)	0.05 (0.00, 0.10)	0.10 (0.05, 0.16)***	0.09 (0.03, 0.16)**

*p ≤ .05, **p ≤ .01, ***p ≤ .001

^a^Binary logistic regression (OR ± 95% CI)

^b^Survey year centred on 1994

^c^A dichotomized category was created ‘being bullied’ (more than 2–3 times a week in the past couple of months) vs ‘non-bullied’

^d^General linear model (B ± 95% CI) (higher values represent more frequent psychological complaints)

### Confidence

The relationship between being bullied and confidence is presented in Fig. 1 (see Supplementary Materials). An increase in feeling highly confident for 11-year-old girls [*F* (1,1453) = 14.28, *p* < .001] was observed, with those who have not been bullied registering an average 2% increase in the likelihood of reporting high confidence every year. On the other hand, a decrease in confidence levels over time was observed for older not bullied adolescent girls. The strongest decrease in confidence for those who were not bullied was seen in 15-year-old girls [*F* (1,1453) = 36.28, *p* < .001]. This group was 3% more likely every year to indicate lower levels of confidence (OR 0.97, 95% CI 0.96–0.98). Apart from the 13- and 15-year-old girls, for all the other age/gender permutations, pupils who were bullied were less likely to report high levels of confidence. The strongest association could be seen for the 15-year-old boys [*F* (1,1453) = 19.10, *p* < .001]. A significant interaction between survey year and being bullied on confidence levels could be seen for girls (all age groups) and 13-year-old boys. Over time bullied pupils show reducing levels of high confidence, relative to non-bullied pupils. The strongest effects could be seen for 13- [*F* (1,1453) = 9.32, *p* < .05] and 15-year-old girls [*F* (1,1453) = 4.82, *p* < .05], such that with every calendar year bully victims were on average 4% less likely to indicate high levels of confidence compared to their non-bullied peers.

### Happiness

Over time a general increase in prevalence of happiness could be seen for non-bullied 11- and 13-year-old boys and girls. For non-bullied girls, the strongest effect was for 11-year-olds [*F* (1,1453) = 18.10, *p* < .001], with every year seeing an average 4% increase in those reporting feeling happy. No increase over time was observed for the 15-year-olds, irrespective of their gender. For all gender/age permutations, in 1994 pupils who were bullied were less likely to report high levels of happiness [*F* (1,1453) = 6.06, *p* < .05] than those who were not bullied. The strongest associations could be seen for the 13-year-old boys [*F* (1,1453) = 53.39, *p* < .001]. An interaction between survey year and bullying victimisation was only seen for 13- [*F* (1,1453) = 9.83, *p* < .001] and 15-year-old girls [*F* (1,1453) = 7.26.11, *p* < .05]. With every calendar year, older adolescent girls who were bullied were on average 4% less likely to indicate high levels of happiness than their non-bullied counterparts. No significant associations emerged for any other gender/age groups.

### Psychological complaints

Between 1994 and 2014, an overall decrease in experiencing psychological complaints was observed for boys who had not been bullied in all age groups. The strongest effect was observed for 11-year-old boys (*β* = −0.07, SE_b_ = 0.001, *t* = −8.79, *p* < .005). However, a divergent pattern by age emerged for girls. For the 11-year-old girls a decrease over time was observed (*β* = −0.07, SE_b_ = 0.01, *t* = −7.21, *p* < .001), whereas for 15-year-olds an increase was seen (*β* = 0.09, SE_b_ = 0.01, *t* = 7.81, *p* < .001). At the first survey, all pupils who were bullied indicated higher levels of psychological complaints. Whilst no interaction between survey year and bullying victimization was seen for boys (all age groups) and 11-year-old girls, a significant association was observed for the 13- (*β* = 0.10, SE_b_ = 0.03, *t* = 3.65, *p* < .001) and 15-year-olds girls (*β* = 0.09, SE_b_ = 0.03, *t* = 2.89, *p* < .05). Relative to those who were not bullied, girls who were bullied became increasingly likely to report psychological complaints since 1994.

## Discussion

This study represents a unique investigation into the changing associations between bullying victimization and mental well-being indicators among a representative sample of young people in Scotland over two decades (1994–2014). Contrary to previous international findings (Chester et al. 2015), an increase in bullying victimization rates in Scotland could be seen over this period, apart from 13-year-old boys and 15-year-old girls. The greatest increase was seen for 13-year-old girls (from 11.3% in 1994 to 18.9% in 2014). Similar findings in other European representative samples indicate a comparable increase in victimization rates, especially for girls (Ilola and Sourander 2013). Our findings also indicated no significant gender difference in bullying victimization, although in survey years there was a significant drop with age. This second finding is in line with previous research showing that frequency of victimization decreases by the end of high school for both genders (Hymel and Swearer 2015).

The overall increase in bullying victimization despite national and local efforts could be explained by several factors. First, it is possible that due to school level initiatives, schoolchildren are more aware of bullying and are thus more ready to recognize and report victimization. Second, in the past decade innovations in technology including the advent of social media has led to a rise in the number of cyber victimization incidents (Haddon and Livingstone 2012). Cross-national studies indicate that on average 3% (HBSC Survey) (Inchley and Currie 2016) or 6% (EU Kids Online Survey) (Haddon and Livingstone 2012) of young people have been victimized though the use of electronic devices. In this context, it could be that the victimization increase is driven by the increasing rates of cyberbullying. While there are studies which indicate that online victims are part of a different cohort compared to those bullied face-to-face, a growing body of research argues that these two groups are actually formed by the same individuals (Kubiszewski et al. 2015).

Similar to previous research, bullied schoolchildren were more likely to report lower levels of confidence and happiness, and higher levels of psychological symptoms. This reflects the fact that being bullied interferes with young people’s emotional and psychological well-being. Moreover, our results suggest that the recently observed worsening of mental well-being could be partially explained by the experience of bullying victimization, especially in the case of adolescent girls. However, due to the cross-sectional nature of the HBSC study, no causality can be implied. In this study, bullying victimization was considered to be a predictor of mental well-being. It is important to note the possible bi-directional nature of the observed associations. Other studies have found that bullying victimization can act both as an antecedent to internalizing problems, and as a consequence of them (Juvonen et al. 2000).

The strongest change in the association between bullying victimization and mental well-being was seen for the older age groups, especially in girls. This effect may be partially explained by the fact that in some cases peer victimization is typically a stable experience (Pouwels et al. 2016); therefore, a cumulative effect in older adolescents could be observed (Hymel and Swearer 2015). Particularly in the case of adolescent girls, previous research suggests that factors such as dysfunctional interaction patterns, interactional styles that are sustained by the progressive accumulation of their own consequences (cumulative continuity model) or insufficient peer support (Caspi et al. 1987) could explain the worsening effect of mental well-being indicators over time. Moreover, a recent review indicates that the association between bullying victimization and internalizing problems was stronger in older adolescents compared to children (Cook et al. 2010). A reduction over time in perceived support (parents, peers, classmates and teacher)—especially for older adolescents (Currie et al. 2015)—could also contribute to the observed patterns. Future studies should seek to explore these possible mechanisms, as well as other moderators in the interplay for bullying victimization and mental well-being.

This study showed a steady increase in bullying victimization rates among Scottish young people in the past two decades. Additionally, our findings indicate that the associations between bullying victimization and mental well-being vary by age and gender. For example, the decline in mental well-being for bullied adolescent girls was particularly acute. A key strength of this study was the large sample size which was representative of Scottish school-aged children over a period of two decades. Furthermore, this study progresses the literature on bullying victimization by revealing how the associations between victimization and mental well-being have changed over time by looking at age and gender differences. Nonetheless, several limits of this study should be mentioned. Although this study collected data over time, the cross-sectional design does not permit the investigation of causal mechanisms. Moreover, all measures used in this study were based on self-report responses, which may be open to particular bias in the reporting of previous bullying experiences. Further, what young people interpret to constitute bullying may have changed over time. However, this risk was minimized due to anonymity of responses, and this potential bias cannot account for the changing trends over time.

Bullying prevention remains a major challenge for educational and public health services in Scotland. Based on the findings presented here, there is a need to focus on targeted intervention in response to bullying victimization. Moreover, it is important to intervene at an age before victimization becomes highly resistant to change (Rueger et al. 2011). Despite the national and local efforts in Scotland, an increase in victimization rates was observed, and this was coupled with worsening mental well-being for victims. This highlights the need for continued efforts to ensure that anti-bullying programmes are effective and support those who are most at risk.

## Electronic supplementary material

Below is the link to the electronic supplementary material.

Supplementary material 1 (DOC 289 KB)
