# Supplementary material for: Trends in bullying victimization in Scottish adolescents 1994–2014: changing associations with mental well-being
Source: Int J Public Health. 2017 Mar 15;62(6):639–46. doi: 10.1007/s00038-017-0965-6 (PMC5487886; doi:10.1007/s00038-017-0965-6)
Supplement: Supplementary file 1 — Supplementary material 1 (DOC 289 KB) [file 38_2017_965_MOESM1_ESM.doc]

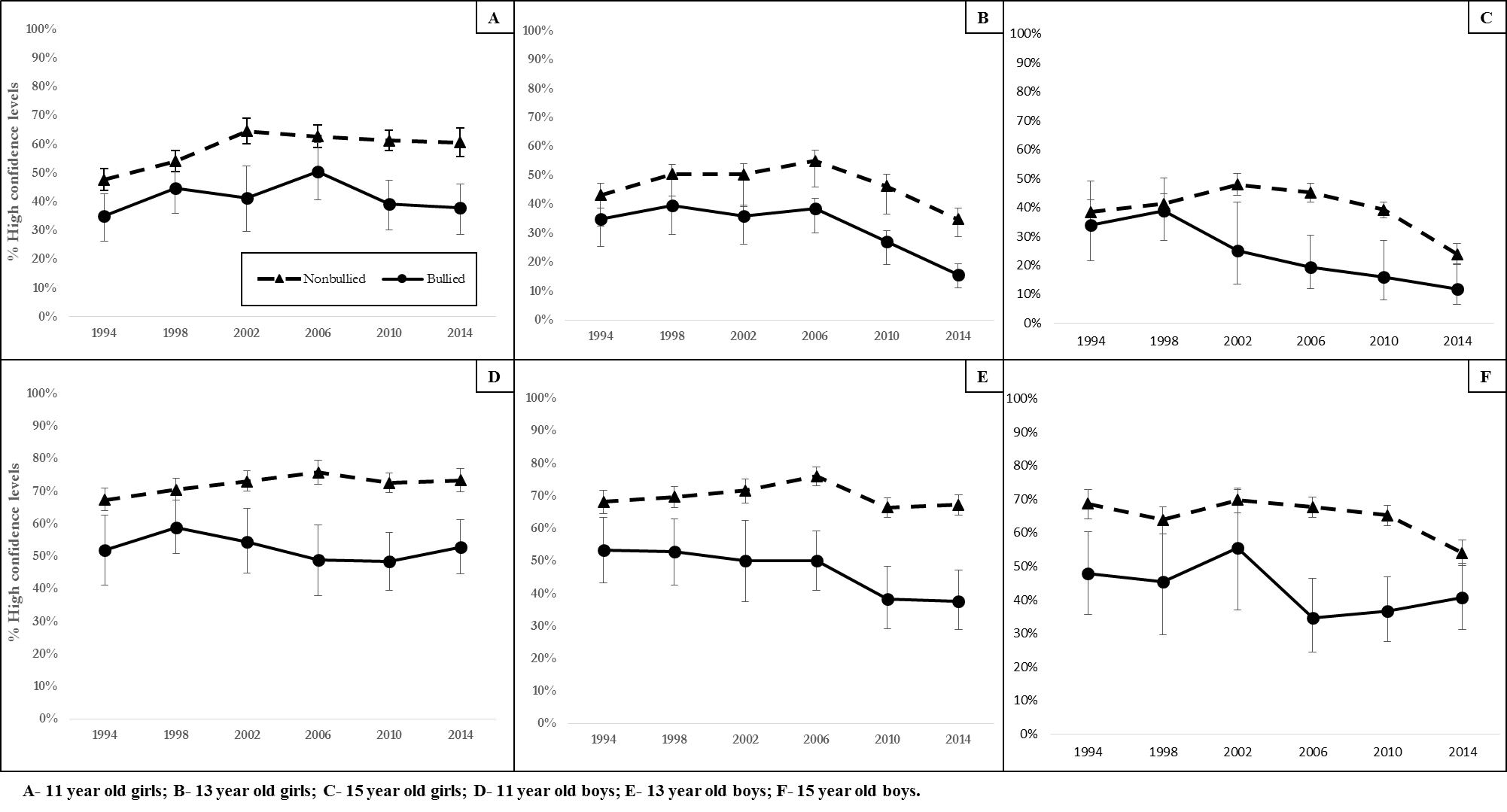


Figure 1 The associations overtime (1994 to 2014) bullying victimization and confidence among Scottish adolescents


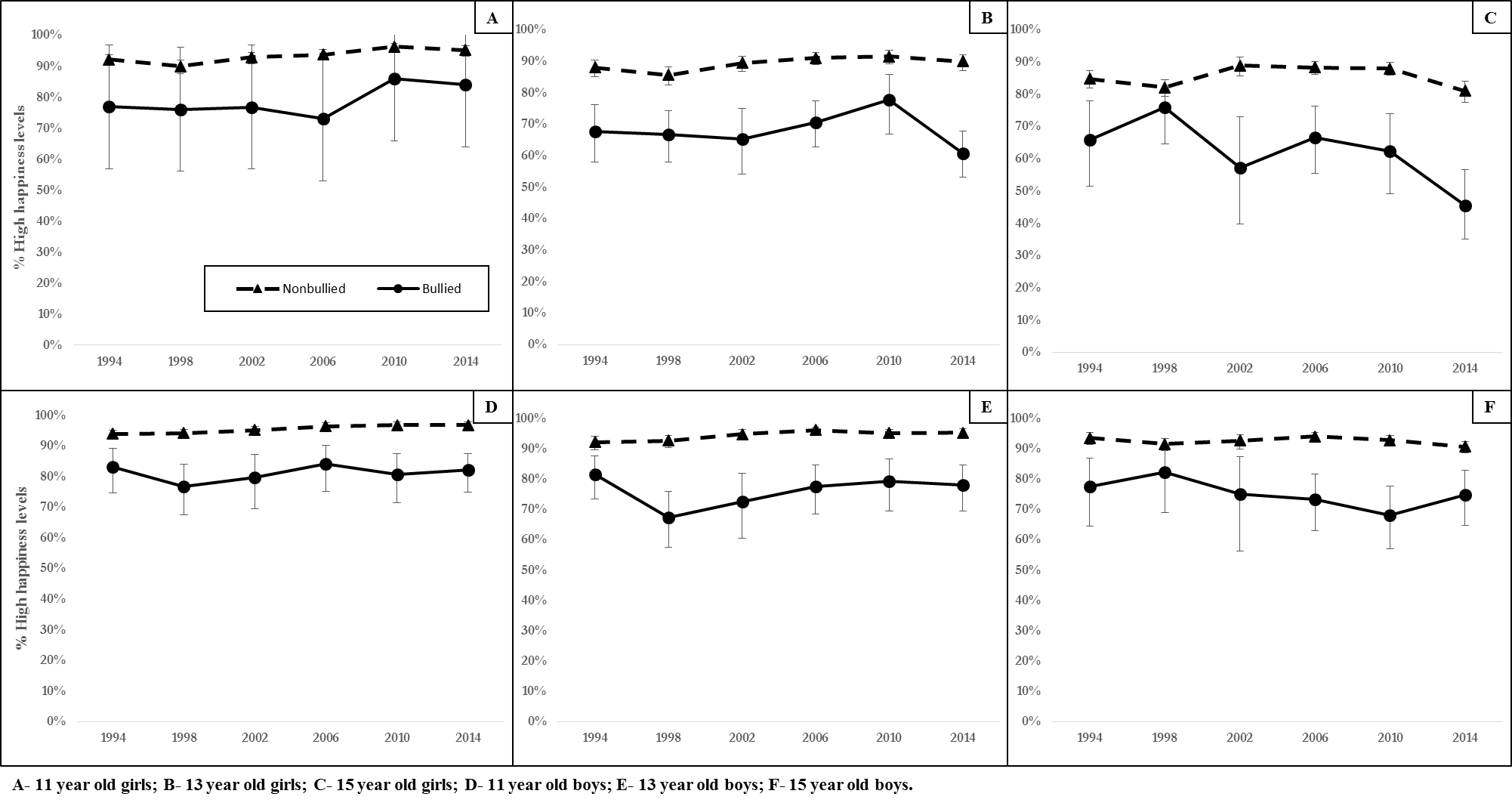


Figure 2 The associations overtime (1994 to 2014) bullying victimization and happiness among Scottish adolescents


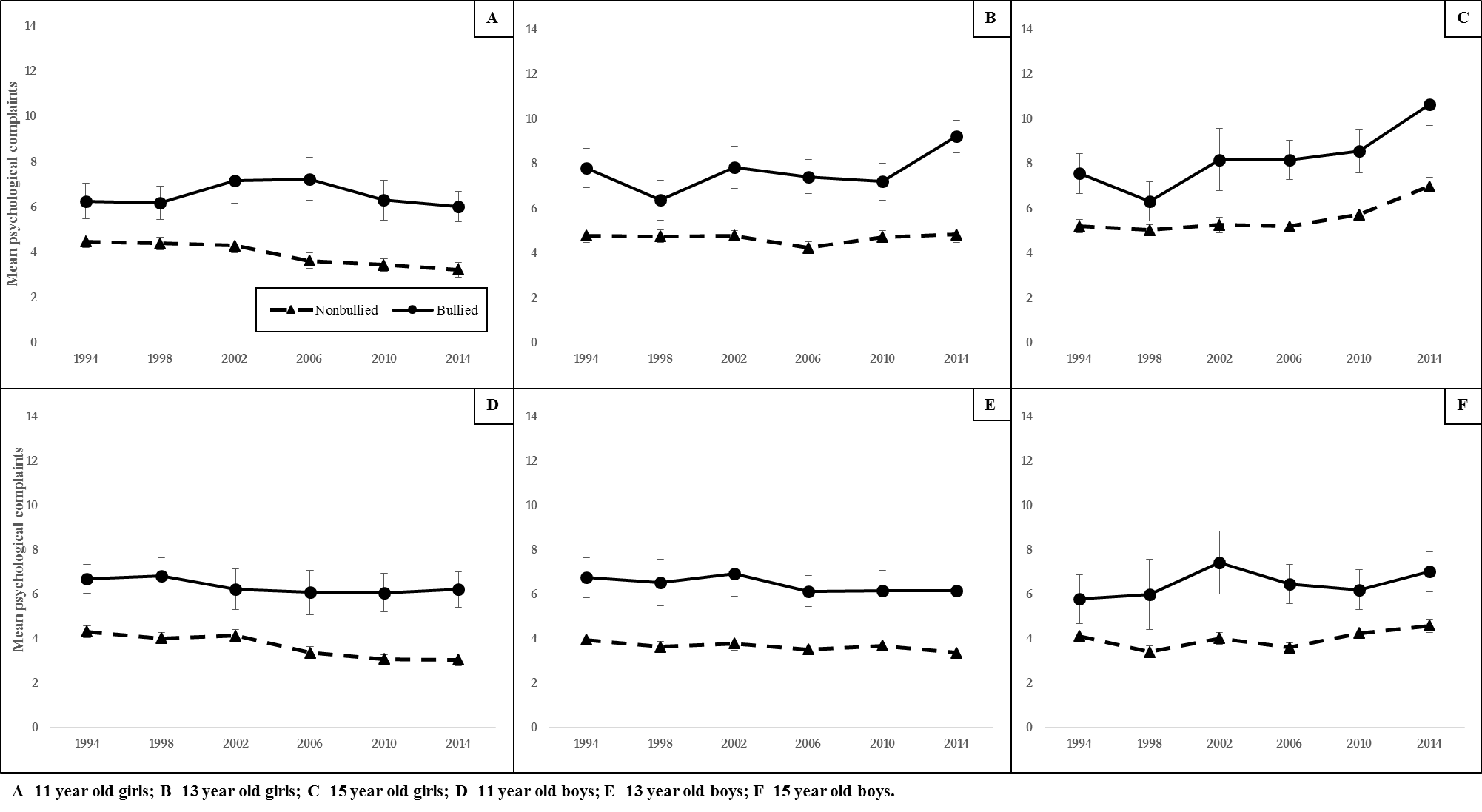


Figure 3 The associations overtime (1994 to 2014) bullying victimization and psychological complaints among Scottish adolescents
